# A Systematic Review of the Relationship Between Host Personality Traits and Parasitic Infection

**DOI:** 10.3390/biology15060490

**Published:** 2026-03-19

**Authors:** Zhu-Cheng Gao, Ling-Ying Shuai, Li-Qing Wang, Meng-Yuan Xu, Dong Yu, Zhi-Tao Liu

**Affiliations:** 1 College of Life Science and Technology, Songbei Campus, Harbin Normal University, Harbin 150025, China; 13644578677@163.com (Z.-C.G.); yudong681101@126.com (D.Y.); 2 College of Life Sciences, Huaibei Normal University, Huaibei 235000, China; shuailingying@163.com (L.-Y.S.); 19856100652@163.com (M.-Y.X.); 3 Grassland Research Institute, Chinese Academy of Agricultural Sciences, Hohhot 010010, China; wangliqing0471@163.com

**Keywords:** activity, boldness, exploration, host behavior, parasitism, personality

## Abstract

Parasitism plays an important role in many fundamental ecological processes and is widespread both geographically and taxonomically. Interactions between parasites and hosts are often complex, and much attention has been paid to the relationship between host traits and parasitism. However, a comprehensive review of the association between host personality and parasitic infection is lacking. In this review, we systematically synthesized 54 studies published over the past few decades. Generally, the relationship between animal personality traits and parasitic infection is complex, and no generic pattern exists. Many biological processes may be involved, and biological factors (such as host sex and parasite type) may significantly shape the personality–infection relationship. Different personality traits may also indicate different relationships with parasitic infection. We confirmed that host personality and parasitic infection exhibit a mutually influential, multi-feedback dynamic. Finally, several research gaps were described, and we emphasized the importance of incorporating the BACI design in experiments.

## Simple Summary

Parasitism is a fundamental type of interspecific interaction, and exploring the mechanisms behind parasitic infection is vital for understanding the role of parasitism in shaping host population dynamics, host–parasite co-evolution, and ecosystem functioning. Previous studies suggested that parasitism is closely related to many traits of both hosts and parasites, and the relationship between host personality traits and parasitic infection has been widely explored in recent decades. This review summarizes main findings of the relevant empirical works. The results of previous studies are complex and context-dependent, with positive, negative, and neutral associations being repeatedly reported. We then analyzed the potential mechanisms behind these patterns, research methods and the limitations of the previous studies, and new directions in future studies were also discussed.

## 1. Introduction

Parasitism is one of the most fundamental forms of interaction in ecosystems, and parasites are remarkably widespread in both taxonomy and geographic distribution [1]. Depending on host-derived nutritional resources for reproduction and survival, parasites establish intimate interspecific associations with their hosts and exert profound effects on the host’s physiological functions, behavioral patterns, survival rates, and population dynamics [2,3,4,5]. To understand this core ecological relationship, research dedicated to unraveling the interaction mechanisms between parasites and hosts has emerged as a prominent field [6], revealing that numerous factors modulate infection, such as host body size, sex differences, and population density [2,3,4,5].

The relationships between host behavior and parasitic infection are particularly pivotal, and nearly all host behaviors are directly linked to the risk of parasite exposure [7,8,9]. For example, mating behavior acts as a key vector for the transmission of bacteria, protozoa, and viruses; foraging represents the primary pathway through which nematodes infect hosts; and social interactions facilitate the dissemination of contact-transmitted parasites. Animals with more pronounced exploration and activity-related behaviors are prone to increased exposure to parasites and thus parasitic infection [10]. To a certain extent, parasites can alter host behavior, which can also modulate the severity of parasitic infection. For example, research has demonstrated that the parasite *Toxoplasma gondii* may manipulate the behavior of rodents by facilitating predation, thereby enhancing its transmission to feline definitive hosts [11].

The term ‘personality’ was first used in anthropological research and is commonly found in psychological literature. In behavioral ecology, however, ‘personality’ refers to consistent and relatively stable behavioral differences among individuals [12,13]. In the late 1970s, Stevenson-Hinde and others published a series of landmark research on animal personality [14]. Based on this, researchers refer to the ‘Big Five Personality Model’ system from psychological studies to create a ‘Five Major Categories Model’ system for animal personality traits [15]. Personality traits can then be summarized into several aspects such as boldness, exploration, activity, aggressiveness and sociality [16,17]. Contemporary research primarily employs three methods for assessing personality traits: rating scales, behavioral coding, and behavioral experiments. The most prevalent approach is constructing specific behavioral experiments (such as open field, elevated plus maze, novel object contact tests) to measure targeted personality dimensions, while controlling environmental variables such as lighting and sound [18]. For instance, boldness and activity are often evaluated by recording the animals’ movements in an open field arena. Aggressiveness and sociability are usually assessed through mirror tests and observations of interspecific interactions.

It has been indicated that a host’s personality reflects different life-history strategies and is closely linked to speciation, genetic variation, and population dynamics [13]. Individuals with different personalities often exhibit significant differences in foraging strategies, space use, or dispersal [19,20,21], which can profoundly influence survival, reproduction, and individual fitness [13]. Given that personality drives individual behavioral variations, and that behavioral pattern is often related to parasite infection [22], it can be inferred that host personality may also be associated with parasitic infection. Meanwhile, parasitic infection may also affect host personality. By exploring the connection between host personality traits and parasitic infection, new perspectives can be provided for wildlife disease prevention and control, as well as parasite management in livestock and poultry farming. In recent decades, a growing number of studies explored the correlation patterns and mechanisms between host personality traits and parasitic infection. However, a systematic review on this topic is still lacking.

In this paper, we systematically overview the main findings of previous studies on relationships between host personality traits and parasite infection. We also aim to discuss the potential mechanisms behind these associations and highlight the key knowledge gaps and methodological limitations that should be considered in study design. Our review should provide useful insights for future studies in related disciplines such as behavioral ecology, parasitology, and evolutionary ecology, as well as practice in wildlife management, animal welfare and disease control.

## 2. Methods

### 2.1. Literature Search and Screening

We conducted a literature search on Web of Science using the keywords ‘personality’ or ‘behavioral syndrome’ and ’parasite’ or ’parasitism’ or ‘ectoparasite’ or ‘endoparasite,’ as well as the English names of various parasitic taxa (such as ‘flea’ or ‘tick’ or ‘nematode’, and so on). A total of 6083 relevant articles were retrieved, which were then screened for those covering the relationship between host personality traits and parasitic infection.

### 2.2. Screening Process

First, the 6083 publications were imported into an Excel spreadsheet using the built-in export function on the Web of Science. The exported data included four key elements: article title, author(s), publication year, and abstract. Each publication was then checked for relevance by reading its title and abstract. A total of 79 publications were retained after this screening process.

### 2.3. Classification

**Figure 1:**
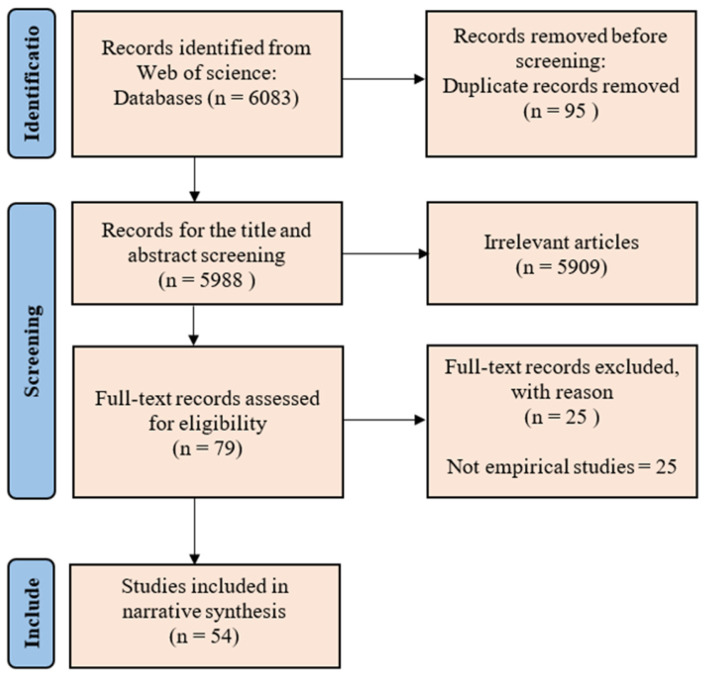
Flowchart of literature screening process.

The 79 publications that remained were then categorized into three types: empirical study, review, and theoretical articles. Reviews and theoretical studies were then excluded. A total of 54 empirical studies were identified, which formed the basis of this review (See Supplementary File Table S1). The literature screening and selection process is illustrated using a PRISMA flow diagram (https://www.prisma-statement.org/, accessed on 27 February 2026) (Figure 1).

## 3. Discussion

### 3.1. Overview

**Figure 2:**
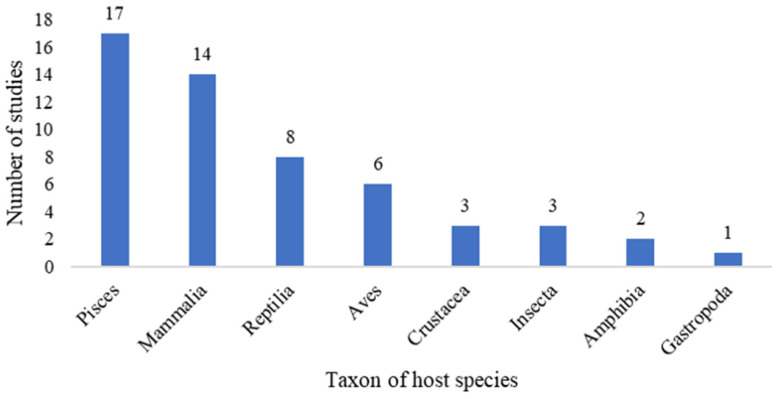
Schematic diagram of host species and the number of studies.

**Figure 3:**
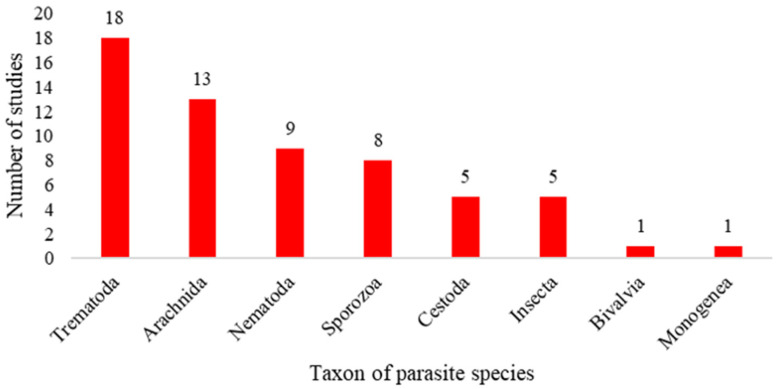
Schematic diagram of parasite species and the number of studies.

The 54 relevant studies covered a wide range of host species, ranging from the structurally simple Gastropoda to the physiologically complex Mammalia. Currently, fish and mammals are the host taxa that receive most attention (Figure 2). The parasites are primarily categorized as ectoparasites (*n* = 20) and endoparasites (*n* = 41). Among them, Trematoda account for the largest proportion, while Bivalvia and Monogenea account for a smaller proportion (Figure 3).

**Figure 4:**
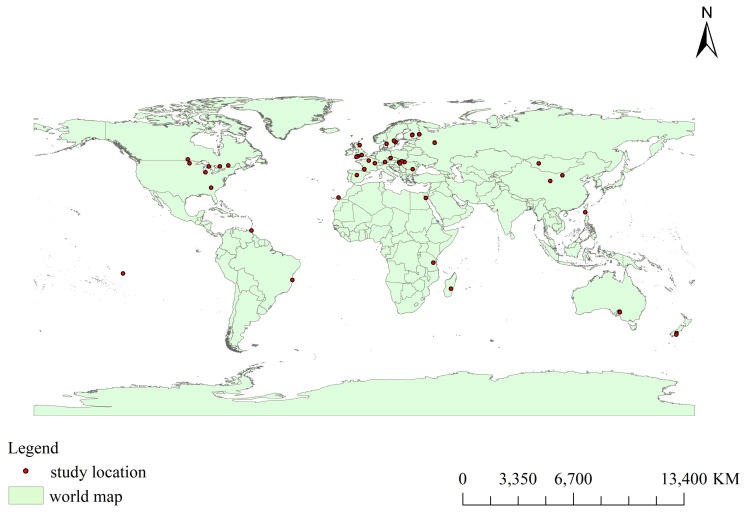
Geographical distribution of relevant studies.

**Figure 5:**
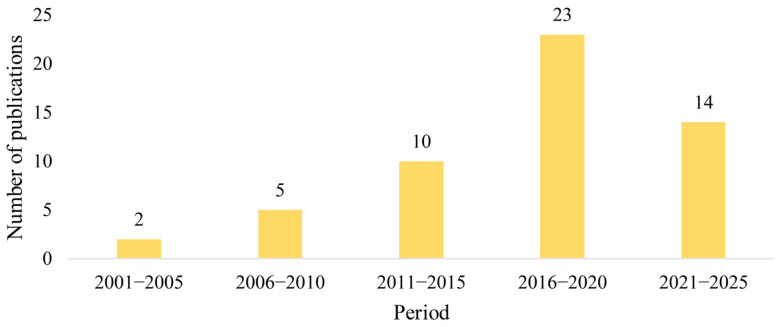
Publication years of relevant studies.

The study locations spanned most continents. In 52 studies, the location was clearly indicated: Europe (*n* = 25), North America (*n* = 8), Oceania (*n* = 11), Africa (*n* = 3), Asia (*n* = 4), and South America (*n* = 1) (Figure 4). Moreover, a five-year interval was adopted as the temporal scale for literature collection. An analysis of the publication years indicated an overall pattern of steady increase in research output, although the number of publications slightly dropped in 2021–2025 (Figure 5).

**Figure 6:**
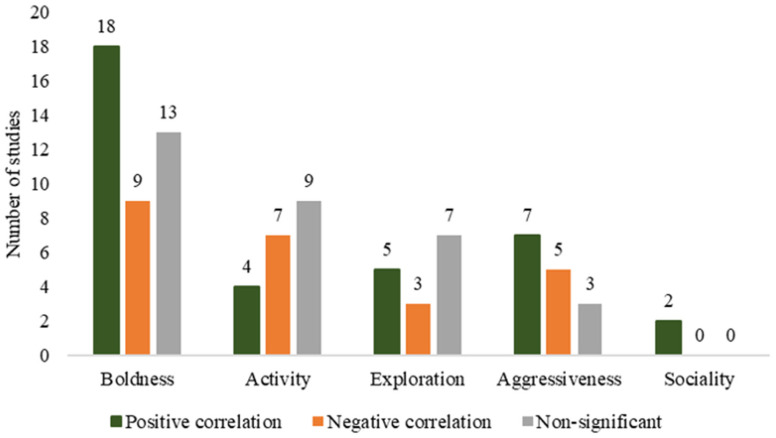
Number of studies covering different traits and their correlations with parasitic infection.

All the five major personality dimensions were explored, but with an uneven distribution of studies: boldness (43.5% of studies), activity (21.7%), exploration (16.3%), aggressiveness (16.3%), and sociality (2.1%) (Figure 6). It is worth noting that some studies investigated the relationship between multiple animal personality traits and parasitic infection.

We detected some significant research biases from previous studies. For example, although a wide range of host species have been involved, the hosts studied are still highly concentrated in vertebrates such as Mammals, Pisces and Reptilia, while much less attention is paid to invertebrate hosts. In terms of the geographical distribution of research, most studies are carried out in developed regions such as Europe, Oceania, and North America (accounting for as much as 84.6% of all the studies), whereas studies in Africa, Asia and South America are relatively rare. This is probably due to availability of research resources (e.g., funds, laboratories and qualified researchers). Moreover, compared to the other four dimensions of personality traits, sociality is much less considered (only 2 study cases), although it seems reasonable to predict that more sociable individuals are more likely to get infected.

### 3.2. Correlation Between Host Personality Traits and Parasitic Infection: No Generic Pattern

A positive relationship between certain personality traits and parasitic infection has been frequently detected in previous studies. For example, in three-spined sticklebacks (*Gasterosteus aculeatus*), there seems to be a significantly positive relationship between their personality traits (boldness, sociality and activity) and parasitic infection with Glugea [23]. Similarly, increasing aggressiveness in mouse lemurs (*Microcebus rufus*) is correlated with heavier parasitic infection [24]. This phenomenon may be influenced by host hormone levels, which modulate degrees of aggressive behavior [25,26]. Ectoparasites, such as lice, can be transmitted through inter-host contact behaviors, including aggressiveness, reproduction, and social interaction. However, whether heavy ectoparasitic infestation can influence host aggressiveness remains an open question.

Not all studies find such a positive correlation. Some studies even obtained opposite results [23,27,28]. For example, the boldness of three-spined sticklebacks (*Gasterosteus aculeatus*) was negatively related to the degree of parasitic infection with *Schistocephalus solidus* [29]. The researchers thoroughly considered and discussed this phenomenon. One possible explanation is based on the differences in circulating hormones (such as cortisol), which could influence both host behavior and immune competence. These findings shift the focus from a simplistic binary relationship between host personality traits and parasitic infection toward a more multifaceted consideration involving factors such as host immunity and physiological mechanisms. This provides new perspectives for future discussions on the relationship between host personality traits and parasitic infection.

The absence of a significant correlation is also worth noting. For instance, a study found that trematode metacercaria infection is not significantly correlated with activity or boldness of nymphs of the red damselfly (*Xanthomis zealandica*) [30]. However, the authors still considered potential relationships between personality traits and parasitic infection, focusing on the discussion and analysis of the experimental design. For example, they proposed that the results may have been affected by the time of infection [31,32,33]. Many larval parasites need to develop within the intermediate host for a certain period of time before they can trigger changes in host behavior and personality traits. Some of the investigated infections may have occurred recently, and thus the parasites did not yet fully develop to alter host behavior. Alternatively, the ability to alter host behavior may be related to the intensity of infection. The hosts in this experiment had low infection levels, which may have been insufficient to affect hosts. In addition, the experimental setup may not be sufficient to detect very subtle behavioral changes, as behavioral changes may only manifest under specific environmental conditions. Finally, it is also possible that the studied trematode was unable to manipulate host behavior at all, as this ability is not expected to evolve or be expressed in all parasite species [34]. Such non-significant relationships have been documented in many studies [35,36,37].

Such mixed results partly reflect the complex interactions between host personality and parasitic infection. On one hand, personality can affect parasitic infection in many ways, and the mechanisms may be either behavioral or physiological. For example, bolder, more active or more explorative individuals may have larger home ranges and more chances to contact other individuals, thus increasing their encounter rate of parasites [38]. Moreover, personality traits and immunities of animals may co-evolve, as individuals exhibiting higher activity or boldness are more likely to have higher immune function and more effective resistance to parasites [39]. On the other hand, parasites may also alter host behavior and even its personality [40]. As a result, complicated feedback loops may arise and it would thus be difficult to predict the overall relationship [22].

### 3.3. The Roles of Ecological Factors in Shaping the Personality–Infection Relationship

Previous studies suggest that the relationship between host personality and parasitic infection is rather complex, and there seems to be no unique pattern. It would be interesting to explore how the other ecological factors shape the host personality-parasitic infection relationship. For instance, certain studies have found that in some species, only females exhibit a significant correlation between personality traits and parasitic infection, whereas males do not [40,41]. This phenomenon raises further questions on whether and how sex plays a role in the relationship between host personality traits and parasitic infection.

As a critically important factor influencing personality traits, sex has been incorporated into research investigating the relationship between personality and parasitic infection. One study found that exploration behavior in mice was positively correlated with *Toxoplasma gondii* infection in female but not male hosts [40]. These differences may be attributed to parasite-induced alterations in the host’s dopaminergic system. The dopaminergic system differs between sexes, and thus sex-based differences affect the significance of the association between personality traits and parasitic infection.

Another study confirmed that female beetle *Odontotaenius disjunctus* with severe parasite infections exhibited greater boldness, whereas male beetle *Odontotaenius disjunctus* with severe parasite infections displayed increased cautious [42]. In contrast to previous research that highlighted a role of a single sex in the relationship between host personality traits and parasitic infection, this study revealed two distinct and significant correlations corresponding to the two sexes. To date, most studies on the physiology and biological functions of *O. disjunctus* have indicated no significant differences between males and females regarding energy usage, basal metabolic rate, feeding rate, heart rate, and immune system performance [43,44,45]. However, the most important indicator of behavioral differences between the sexes was their response to dangerous environments in the absence of parasitic infection. This study found that in uninfected groups, male and female beetles may exhibit inherently different strategies when confronted with stressors. These different coping mechanisms indirectly influence the relationship between personality traits and parasitic infection. This outcome confirms that sex influences the interplay between personality traits and parasitic infection: Although sex does not alter the fundamental nature of the interaction, it can modulate the direction of its effect.

Differences in host species, parasite type and personality dimensions considered may also affect the association between host personality and parasitic infection. For example, parasite infection was positively related to the boldness of *Lepomis gibbosus*, but negatively correlated with its activity level [46]. Such studies also reveals the complexity of the relationship between personality and parasite infection from the perspective of within-individual variation: different personality dimensions of the same host species can exhibit differential associations with parasite infection through behavioral patterns, external environment, energy allocation, and immune trade-offs [47]. Moreover, the level of interaction with host behavior may also differ between parasite types (e.g., parasites that reproduce within hosts and those that do not), which deserve further investigations.

### 3.4. Correlation or Causality?

A study conducted at Bundey Bore Station in South Australia on the interaction between wild lizards and parasites found that among less aggressive lizards, higher boldness was associated with lower infection rates, whereas the opposite was true for more aggressive individuals. Additionally, tick infection did not alter the hosts’ inherent aggressiveness, but rather increased their boldness [48]. This case suggests that although personality influences infection, parasites may also significantly reshape hosts’ personality traits. Since these two processes may take effect in a single study, it is not always easy to infer the causal relationship between host personality and parasitic infection. Despite that many researchers have attempted to control the confounding effects of other factors, the results of most studies are still only correlational by essence. For instance, although some researchers experimentally divided the experimental individuals into infected and non-infected groups, they did not perform personality trait tests on the individuals prior to infection [23,49]. As a result, it would be difficult to elucidate the causal relationship between host personality and parasitic infection.

Generally, experiments based on a before-after-control-intact (BACI) design are more powerful to verify the causal relationship studied. In our case, it is important to ensure that the experimental individuals are uninfected at the beginning of the experiment. If the purpose of the experiment is to verify the effect of host personality traits on parasitic infection, then the personality traits of uninfected individuals should be tested first. After this, the infection experiment should be conducted, and the infection status of individuals with different personality traits should be compared. If the purpose of the experiment is to verify the effect of parasitic infection on host personality traits, then the uninfected individuals should be tested for personality and then randomly divided into two or more groups. Different groups should then be subject to different levels of experimental infection (e.g., not infected, slightly infected and heavily infected). After this procedure, all the individuals should be tested for personality once again. Only through such experimental design, could we accurately detect the causal relationship between host personality traits and parasitic infection. A study on *Oncorhynchus mykiss* provides an excellent example [50]. The experimental individuals used in the study were all uninfected state at first and then tested for personality traits before being infected with parasites. Two follow-up personality tests were conducted on the 16th and 38th days after infection with parasites, respectively. Unfortunately, among the 54 studies analyzed here, only 4 studies (7.4%) were based on such a BACI procedure (See Supplementary File Table S2).

## 4. Conclusions

In conclusion, the relationship between animal personality traits and parasitic infection is complex, and it seems that no generic pattern exists. Many biological processes may be involved, and some biological factors may significantly shape the personality–infection relationship. We advocate that experiments based on BACI design should be more widely used in future studies, and more attention should be paid to less studied taxa, regions and dimensions of personality traits. A deeper understanding of the mechanisms underlying the personality–infection relationship would help to understand the mechanisms behind evolution and maintenance of behavioral diversity. In practice, progress in this field is crucial for predicting disease transmission dynamics and developing anti-parasitic drug or targeted therapeutics.

## Data Availability

No new data were created or analyzed in this study. Data sharing is not applicable to this article.

## Supplementary Materials

The following supporting information can be downloaded at: https://www.mdpi.com/article/10.3390/biology15060490/s1, Table S1. Summary of articles retained after final screening, Table S2. Comprehensive overview of the literature.

## References

[1] Carlson C.J., Hopkins S., Bell K.C., Doña J., Godfrey S.S., Kwak M.L., Lafferty K.D., Moir M.L., Speer K.A., Strona G. (2020). A global parasite conservation plan. Biol. Conserv..

[2] Patterson B.D., Dick C.W., Dittmar K. (2008). Parasitism by bat flies (Diptera: Streblidae) on neotropical bats: Effects of host body size, distribution, and abundance. Parasitol. Res..

[3] Postawa T., Nagy Z. (2016). Variation of parasitism patterns in bats during hibernation: The effect of host species, resources, health status, and hibernation period. Parasitol. Res..

[4] Li K., Wang Z.-X., Zhang L., Lun X.-C., Shang M., Xu L., Zhao N., Liu Q.-Y. (2024). The influence of meteorological factors and flea index on the population density of *Meriones unguiculatus* in Xilingol League, Inner Mongolia Autonomous Region from 2012 to 2021. Chin. J. Media Biol. Control.

[5] Zhang X.-Z., Huang X.-B., Wang Y.-J., Yang J.-T., Zheng X.-Y. (2023). Study on the infection and influencing factors of parasites on the surface of southern bats in Yunnan Province. Chin. J. Zoonoses.

[6] Bielby J., Price S.J., Monsalve-CarcaÑo C., Bosch J. (2021). Host contribution to parasite persistence is consistent between parasites and over time, but varies spatially. Ecol. Appl..

[7] Moore J. (2002). Parasites and the Behavior of Animals.

[8] Thrall P.H., Antonovics J., Dobson A.P. (2000). Sexually transmitted diseases in polygynous mating systems: Prevalence and impact on reproductive success. Proc. R. Soc. Lond. Ser. B Biol. Sci..

[9] Altizer S., Nunn C.L., Thrall P.H., Gittleman J.L., Antonovics J., Cunningham A.A., Dobson A.P., Ezenwa V., Jones K.E., Pedersen A.B. (2003). Social organization and parasite risk in mammals: Integrating theory and empirical studies. Annu. Rev. Ecol. Evol. Syst..

[10] Sih A., Spiegel O., Godfrey S., Leu S., Bull C.M. (2018). Integrating social networks, animal personalities, movement ecology and parasites: A framework with examples from a lizard. Anim. Behav..

[11] Pardo Gil M., Hegglin D., Briner T., Ruetten M., Müller N., Moré G., Frey C.F., Deplazes P., Basso W. (2023). High prevalence rates of Toxoplasma gondii in cat-hunted small mammals—Evidence for parasite induced behavioural manipulation in the natural environment?. Int. J. Parasitol. Parasites Wildl..

[12] Gosling S.D. (2001). From mice to men: What can we learn about personality from animal research?. Psychol. Bull..

[13] Wolf M., Weissing F.J. (2012). Animal personalities: Consequences for ecology and evolution. Trends Ecol. Evol..

[14] Stevenson-Hinde J., Stillwell-Barnes R., Zunz M. (1980). Individual differences in young rhesus monkeys: Consistency and change. Primates.

[15] Li S., Zhang D., Duan M. (2022). Animal personality and behavior sets: Concepts, testing and analysis. Acta Hydrobiol. Sin..

[16] Réale D., Reader S., Sol D., McDougall P., Dingemanse N. (2007). Integrating animal temperament within ecology and evolution. Biol. Rev..

[17] Fülöp A., Németh Z., Kocsis B., Deák-Molnár B., Bozsoky T., Barta Z. (2019). Personality and social foraging tactic use in free-living Eurasian tree sparrows (*Passer montanus*). Behav. Ecol..

[18] Carter A.J., Feeney W.E., Marshall H.H., Cowlishaw G., Heinsohn R. (2013). Animal personality: What are behavioural ecologists measuring?. Biol. Rev. Camb. Philos. Soc..

[19] Dingemanse N.J., Both C., van Noordwijk A.J., Rutten A.L., Drent P.J. (2003). Natal dispersal and personalities in great tits (*Parus major*). Proc. R. Soc. Lond. Ser. B Biol. Sci..

[20] Mella V.S.A., Ward A.J.W., Banks P.B., McArthur C. (2015). Personality affects the foraging response of a mammalian herbivore to the dual costs of food and fear. Oecologia.

[21] Serrano-Davies E., O’Shea W., Quinn J.L. (2017). Individual foraging preferences are linked to innovativeness and personality in the great tit. Behav. Ecol. Sociobiol..

[22] Ezenwa V.O., Archie E.A., Craft M.E., Hawley D.M., Martin L.B., Moore J., White L. (2016). Host behaviour–parasite feedback: An essential link between animal behaviour and disease ecology. Proc. R. Soc. B Biol. Sci..

[23] Petkova I., Abbey-Lee R.N., Løvlie H. (2018). Parasite infection and host personality: Glugea-infected three-spined sticklebacks are more social. Behav. Ecol. Sociobiol..

[24] Zohdy S., Bisanzio D., Tecot S., Wright P.C., Jernvall J. (2017). Aggression and hormones are associated with heterogeneity in parasitism and parasite dynamics in the brown mouse lemur. Anim. Behav..

[25] Korte S.M., Koolhaas J.M., Wingfield J.C., McEwen B.S. (2005). The Darwinian concept of stress: Benefits of allostasis and costs of allostatic load and the trade-offs in health and disease. Neurosci. Biobehav. Rev..

[26] Wingfield J., Ball G., Dufty A., Hegner R., Ramenofsky M. (1987). Testosterone and aggression in birds. Alfred Dufty.

[27] Slivko V.M., Zhokhov A.E., Gopko M.V., Mikheev V.N. (2021). Agonistic Behavior of young perch *Perca fluviatilis*: The effects of fish size and macroparasite load. J. Ichthyol..

[28] Seaman B., Briffa M. (2015). Parasites and personality in periwinkles (*Littorina littorea*): Infection status is associated with mean-level boldness but not repeatability. Behav. Process..

[29] Yaqub S.Z. (2018). Consequences of Host Personality and Environmental Change for Parasite Infections in Freshwater Fish. Ph.D. Dissertation.

[30] Filion A., Lagrue C., Presswell B., Poulin R. (2017). Behavioural modification of personality traits: Testing the effect of a trematode on nymphs of the red damselfly *Xanthocnemis zealandica*. Parasitol. Res..

[31] Bethel W.M., Holmes J.C. (1974). Correlation of development of altered evasive behavior in gammarus lacustris (*Amphipoda*) harboring cystacanths of polymorphus paradoxus (*Acanthocephala*) with the infectivity to the definitive host. J. Parasitol..

[32] Urdal K., Tierney J.F., Jakobsen P.J. (1995). The Tapeworm Schistocephalus solidus alters the activity and response, but not the predation susceptibility of infected copepods. J. Parasitol..

[33] Robb T., Reid M.L. (1996). Parasite-induced changes in the behaviour of cestode-infected beetles: Adaptation or simple pathology?. Can. J. Zool..

[34] Poulin R., Brockmann H.J., Roper T.J., Naguib M., Wynne-Edwards K.E., Mitani J.C., Simmons L.W. (2010). Chapter 5—Parasite manipulation of host behavior: An update and frequently asked questions. Advances in the Study of Behavior.

[35] Jing C.-L., Lou Y.-Q., Liu H., Song K., Fang Y., Höglund J., Halvarsson P., Sun Y.-H. (2023). Avian malaria parasite infections do not affect personality in the chestnut thrush (*Turdus rubrocanus*) on the Qinghai-Tibet Plateau. Heliyon.

[36] Kekäläinen J., Lai Y.-T., Vainikka A., Sirkka I., Kortet R. (2014). Do brain parasites alter host personality?—Experimental study in minnows. Behav. Ecol. Sociobiol..

[37] Barnard C.J., Sayed E., Barnard L.E., Behnke J.M., Abdel Nabi I., Sherif N., Shutt A., Zalat S. (2003). Local variation in helminth burdens of Egyptian spiny mice (*Acomys cahirinus dimidiatus*) from ecologically similar sites: Relationships with hormone concentrations and social behaviour. J. Helminthol..

[38] Boyer N., Reale D., Marmet J., Pisanu B., Chapuis J.L. (2010). Personality, space use and tick load in an introduced population of Siberian chipmunks *Tamias sibiricus*. J. Anim. Ecol..

[39] Koolhaas J.M. (2008). Coping style and immunity in animals: Making sense of individual variation. Brain Behav. Immun..

[40] SkallovÁ A., Kodym P., Frynta D., Flegr J. (2006). The role of dopamine in Toxoplasma-induced behavioural alterations in mice: An ethological and ethopharmacological study. Parasitology.

[41] Dunn J.C., Cole E.F., Quinn J.L. (2011). Personality and parasites: Sex-dependent associations between avian malaria infection and multiple behavioural traits. Behav. Ecol. Sociobiol..

[42] Davis A.K., Ladd R.R.E., Smith F., Shattuck A. (2023). Sex-specific effects of a parasite on stress-induced freezing behavior in a natural beetle-nematode system. PLoS ONE.

[43] Davis A.K., Calderon L., Lefeuvre J., Sims S., Pearce J., Prouty C. (2020). Healing while parasitized: Impact of a naturally-occurring nematode during energy-intensive wound-healing in a beetle. Physiol. Entomol..

[44] Davis A.K., Vasquez D., LeFeuvre J., Sims S., Craft M., Vizurraga A. (2017). Parasite manipulation of its host’s physiological reaction to acute stress: Experimental results from a natural beetle-nematode system. Physiol. Biochem. Zool..

[45] Davis A.K., Coogler B., Johnson I. (2017). The heartrate reaction to acute stress in horned passalus beetles (*Odontotaenius disjunctus*) is negatively affected by a naturally-occurring nematode parasite. Insects.

[46] Gradito M., Dubois F., Noble D.W.A., Binning S.A. (2024). Double trouble: Host behaviour influences and is influenced by co-infection with parasites. Anim. Behav..

[47] MacKay R.N., Moore P.A. (2021). Parasites differentially impact crayfish personality in different contexts. Behaviour.

[48] Payne E., Sinn D.L., Spiegel O., Gardner M.G., Sih A. (2024). A field experiment reveals reciprocal effects of host personality and parasitism in wild lizards. Behav. Ecol..

[49] Fox A., Hudson P.J. (2001). Parasites reduce territorial behaviour in red grouse (*Lagopus lagopus scoticus*). Ecol. Lett..

[50] Klemme I., Kortet R., Karvonen A. (2016). Parasite infection in a central sensory organ of fish does not affect host personality. Behav. Ecol..

