# Supplementary material for: A Systematic Review of the Relationship Between Host Personality Traits and Parasitic Infection
Source: Biology (Basel). 2026 Mar 19;15(6):490. doi: 10.3390/biology15060490 (PMC13024135; doi:10.3390/biology15060490)
Supplement: Supplementary file 1 [file biology-15-00490-s001.zip › biology-4189932-supplementary.pdf]

Supplementary Materials

Table S1 Summary of articles retained after final screening.

Study ID	Author	Journal	Year	Article Title
1	Fox A, Hudson PJ.	Ecology Letters	2001	Parasites reduce territorial behaviour in red grouse (Lagopus lagopus scoticus)
2	Barnard CJ, Sayed E, Barnard LE, et al.	Journal Of Helminthology	2003	Local variation in helminth burdens of Egyptian spiny mice (Acomys cahirinus dimidiatus) from ecologically similar sites: relationships with hormone concentrations and social behaviour
3	Skallová A, Kodym P, Frynta D, et al.	Parasitology	2006	The role of dopamine in Toxoplasma-induced behavioural alterations in mice: an ethological and ethopharmacological study
4	Boyer N, Réale, D, Marmet J, et al.	Journal of Animal Ecology	2010	Personality, space use and tick load in an introduced population of Siberian chipmunks Tamias sibiricus
5	Richards EL.	Cardiff University (United Kingdom)	2010	Foraging, personality and parasites investigations into the behavioural ecology of fishes
6	Pellegrini AFA, Wisenden BD, Sorensen PW.	Behavioral Ecology And Sociobiology	2010	Bold minnows consistently approach danger in the field and lab in response to either chemical or visual indicators of predation risk
7	Mikheev VN, Pasternak AF, Taskinen J, et al.	Parasites & Vectors	2010	Parasite-induced aggression and impaired contest ability in a fish host
8	Lucy DP, Albrecht ISH.	Animal Behaviour	2011	Behavioural correlates of parasitism and reproductive success in male eastern chipmunks, Tamias striatus
9	Dunn JC, Cole EF, Quinn JL.	Behavioral Ecology and Sociobiology	2011	Personality and parasites: sex-dependent associations between avian malaria infection and multiple behavioural traits
10	Hammond-Tooke CA, Nakagawa S, Poulin R.	Behaviour	2012	Parasitism and behavioural syndromes in the fish Gobiomorphus cotidianus
11	Koprivnikar J, Gibson CH, Redfern JC.	Proceedings of The Royal Society B-Biological Sciences	2012	Infectious personalities: behavioural syndromes and disease risk in larval amphibians
12	Kekäläinen J, Lai YT, Vainikka A, et al.	Behavioral Ecology and Sociobiology	2014	Do brain parasites alter host personality? — Experimental study in minnows
13	Bajer K, Horvath G, Molnar O, et al.	Behavioural Processes	2015	European green lizard (Lacerta viridis) personalities: linking behavioural types to

				ecologically relevant traits at different ontogenetic stages
14	Garamszegi LZ, Zagalska-Neubauer M, Canal D, et al.	Behavioral Ecology	2015	Malaria parasites, immune challenge, MHC variability, and predator avoidance in a passerine bird
15	Kortet R, Sirkka I, Lai YT, et al.	Frontiers in Ecology and Evolution	2015	Personality differences in two minnow populations that differ in their parasitism and predation risk
16	Reisinger LS, Petersen I, Hing, JS, et al.	Freshwater Biology	2015	Infection with a trematode parasite differentially alters competitive interactions and antipredator behaviour in native and invasive crayfish
17	Ben S, Mark B.	Behavioural Processes	2015	Parasites and personality in periwinkles (Littorina littorea): infection status is associated with mean-level boldness but not repeatability
18	Horváth G, Martín J, López P, et al.	Ethology	2016	Blood parasite infection intensity covaries with risk-taking personality in male carpetan rock lizards (Iberolacerta cyreni)
19	Gyuris E, Hankó JF, Feró O, et al.	Behavioural Processes	2016	Personality and ectoparasitic mites (Hemipteroseius adleri) in firebugs (Pyrrhocoris apterus)
20	Pan T, Gladen K, Duncan EC, et al.	Zebrafish	2016	Bold, sedentary fathead minnows have more parasites
21	Wengström N, Wahlqvist F, Näslund J, et al.	Ethology	2016	Do individual activity patterns of brown trout (Salmo trutta) alter the exposure to parasitic freshwater pearl mussel (Margaritifera margaritifera) larvae?
22	Klemme I, Karvonen A.	Proceedings of the Royal Society B-Biological Sciences	2016	Learned parasite avoidance is driven by host personality and resistance to infection in a fish–trematode interaction
23	Klemme I, Kortet R, Karvonen A.	Behavioral Ecology	2016	Parasite infection in a central sensory organ of fish does not affect host personality
24	Zohdy S, Bisanzio D, Tecot S, et al.	Animal Behaviour	2017	Aggression and hormones are associated with heterogeneity in parasitism and parasite dynamics in the brown mouse lemur
25	Bohn S, Webber QMR, Florko KRN, et al.	Ethology	2017	Personality predicts ectoparasite abundance in an asocial sciurid
26	Marinov MP, Zehtindjiev P, Dimitrov D, et al.	Ethology Ecology & Evolution	2017	Haemosporidian infection and host behavioural variation: a case study on wild-caught nightingales (Luscinia megarhynchos)

27	Filion A, Lagrue C, Presswell B, et al.	Parasitology Research	2017	Behavioural modification of personality traits: testing the effect of a trematode on nymphs of the red damselfly Xanthocnemis zealandica
28	Flink H, Behrens JW, Svensson PA.	Parasitology Research	2017	Consequences of eye fluke infection on anti-predator behaviours in invasive round gobies in Kalmar Sound
29	Sih A, Spiegel O, Godfrey S, et al.	Animal Behaviour	2018	Integrating social networks, animal personalities, movement ecology and parasites: a framework with examples from a lizard
30	Piquet JC, López-Darias M, van der Marel A, et al.	Behavioral Ecology and Sociobiology	2018	Unraveling behavioral and pace-of-life syndromes in a reduced parasite and predation pressure context: personality and survival of the Barbary ground squirrel
31	Yaqub S.	University of Leicester (United Kingdom)	2018	Consequences of host personality and environmental change for parasite infection in freshwater fish
32	Petkova I, Abbey-Lee RN, Lovlie H.	Behavioral Ecology and Sociobiology	2018	Parasite infection and host personality: Glugea-infected three-spined sticklebacks are more social
33	Finnerty PB, Shine R, Brown GP.	Functional Ecology	2018	The costs of parasite infection: Effects of removing lungworms on performance, growth and survival of free-ranging cane toads
34	Payne EM.	University of California	2019	Integrating consistent among-individual differences in behavior and parasite load in a wild population of Sleepy Lizards, Tiliqua rugosa
35	Santicchia F, Romeo C, Ferrari N, et al.	Mammalian Biology	2019	The price of being bold? Relationship between personality and endoparasitic infection in a tree squirrel
36	Mikheev V N, Pasternak A F, Taskinen J.	Doklady Biological Sciences	2019	Personality influences risk of parasitism in fish
37	Payne E, Sinn DL, Spiegel O, et al.	Oikos	2020	Consistent individual differences in ecto-parasitism of a long-lived lizard host
38	Santicchia F, Wauters LA, Piscitelli AP, et al.	Journal of Animal Ecology	2020	Spillover of an alien parasite reduces expression of costly behaviour in native host species
39	Costa TSO, Nogueira-FSLG, De Vleeschouwer KM, et al.	Amrrican Journal Of Primatology	2020	Individual behavioral differences and health of golden-headed lion tamarins (Leontopithecus chrysomelas)
40	Ruehle BP, Poulin R.	Behaviour	2020	Potential multidimensional behavioural impacts of differential infection in two fish populations

41	Payne E, Sinn DL, Spiegel O, et al.	Animal Behaviour	2021	Consistent after all: behavioural repeatability in a long-lived lizard across a 6-year field study
42	Vanden Broecke B , Bernaerts L, Ribas A, et al.	Frontiers in Veterinary Science	2021	Linking behavior, co-infection patterns, and viral infection risk with the whole gastrointestinal helminth community structure in Mastomys natalensis
43	Rollins RE, Mouchet A, Margos G, et al.	Behavioral Ecology and Sociobiology	2021	Repeatable differences in exploration behaviour predict tick infestation probability in wild great tits
44	Slivko VM, Zhokhov AE, Gopko MV, et al.	Journal of Ichthyology	2021	Agonistic behavior of young perch perca fluviatilis:The effects of fish size and macroparasite load
45	MacKay RN, Moore PA	Behaviour	2021	Parasites differentially impact crayfish personality in different contexts
46	Perez G.	Folia Parasitologica	2022	Role of bank vole (Myodes glareolus) personality on tick burden (Ixodes spp.)
47	MacKay RN.	Bowling Green State University	2022	Parasites alter organismal behavior and interactions in aquatic ecosystems
48	Jing CL, Lou YQ, Liu H, et al.	Heliyon	2023	Avian malaria parasite infection do not affect personality in the chestnut thrush (Turdus rubrocanus) on the Qinghai-Tibet Plateau
49	Davis AK, Ladd RRE, Smith F, et al.	Plos One	2023	Sex-specific effects of a parasite on stress-induced freezing behavior in a natural beetle-nematode system
50	Payne E, Sinn DL, Spiegel O, et al.	Behavioral Ecology	2024	A field experiment reveals reciprocal effects of host personality and parasitism in wild lizards
51	Eric P, David LS, Orr S, et al.	Ecological Monographs	2024	Personality, space use, and networks directly and indirectly explain tick in festation in a wild population of lizards
52	Kuo YJ, Lee YF, Kuo YM, et al.	Integrative Organismal Biology	2024	Sex and State-Dependent Effects on Proactive Behaviors of Bent-Wing Bats Across Contexts
53	Gradito M, Dubois F, Noble DWA, et al.	Animal Behaviour	2024	Double trouble: host behaviour influences and is influenced by co-infection with parasites
54	Wang R, Wang S, Qu JP.	Peerj	2025	Parasites influence the physiology and personality in a small mammal (Ochotona curzoniae)

Table S2. Comprehensive overview of the literature.

Study ID	Host Name	Host Type	Parasite Name	Parasite Type	Region	Personality-Parasitism Correlation	Infection Metric	Study Type
1	*Tiliqua rugosa*	Reptilia	Tick	Ectoparasite	Oceania	Boldness: negative; Boldness+Aggression: positive	Parasite load	Correlational
2	*Tiliqua rugosa*	Reptilia	Tick	Ectoparasite	Oceania	Aggression: negative	Parasite load	Correlational
3	*Tiliqua rugosa*	Reptilia	Tick	Ectoparasite	Oceania	Boldness/Aggression: negative	Parasite load	Correlational
4	*Iberolacerta cyreni*	Reptilia	Eimeria	Endoparasite	Europe	Boldness: positive	Parasite load	Correlational
5	*Tiliqua rugosa*	Reptilia	Tick	Ectoparasite	Oceania	Boldness/Aggression: positive	Parasite load	Correlational
6	*Tiliqua rugosa*	Reptilia	Tick	Ectoparasite	Oceania	Boldness: positive; Aggression: positive	Parasite load	Correlational
7	*Lacerta viridis*	Reptilia	Tick	Ectoparasite	Europe	exploration: negative	Infection status	Correlational
8	*Tiliqua rugosa*	Reptilia	Tick	Ectoparasite	Oceania	Boldness: positive	Infection intensity	Correlational
9	*Microcebus rufus*	Mammalia	Lice/Nematode	Ecto/Endoparasite	Africa	Aggression: positive	Parasite load	Correlational
10	*Tamias striatus*	Mammalia	Tick/Flea/Mite/Botfly/Tapeworm/Nematode	Ecto/Endoparasite	N. America	Boldness: positive	Parasite load	Correlational
11	*Mastomys natalensis*	Mammalia	Nematode	Endoparasite	Africa	exploration: negative	Parasite load	Correlational
12	*Tamias minimus*	Mammalia	Flea/Mite/Lice	Ectoparasite	N. America	exploration: positive	Parasite load	Correlational

Study ID	Host Name	Host Type	Parasite Name	Parasite Type	Region	Personality-Parasitism Correlation	Infection Metric	Study Type
13	*Tamias sibiricus*	Mammalia	Tick	Ectoparasite	Europe	Activity/exploration: no significant correlation	Parasite load	Correlational
14	*Myodes glareolus*	Mammalia	Tick	Ectoparasite	Europe	Boldness/Activity/exploration: no significant correlation	Parasite load	Correlational
15	*Sciurus vulgaris*	Mammalia	Nematode	Endoparasite	Europe	Activity: negative (red squirrels only)	Infection status	Correlational
16	*Sciurus carolinensis*	Mammalia	Nematode	Endoparasite	Europe	Boldness/exploration: positive	Infection intensity	Correlational
17	*Mus musculus*	Mammalia	Toxoplasma	Endoparasite	Europe	Activity: negative; Exploration (♀): positive	Infection status	Correlational
18	*Atlantoxerus getulus*	Mammalia	Lice	Ectoparasite	Africa	Boldness/exploration: no significant correlation	Parasite load	Correlational
19	*Leontopithecus chrysomelas*	Mammalia	Not mentioned	Endoparasite	S. America	sociality: positive	Parasite load	Correlational
20	*Acomys cahirinus dimidiatus*	Mammalia	Nematode	Endoparasite	Asia	Aggression: no significant correlation	Parasite load	Correlational
21	*Ochotona curzoniae*	Mammalia	Eimeria	Endoparasite	Asia	Boldness/exploration: positive; Aggression: negative	Parasite load	Correlational
22	*Miniopterus fuliginosus*	Mammalia	Batfly	Ectoparasite	Asia	Boldness/Activity/exploration: no significant correlation	Parasite load	Correlational

Study ID	Host Name	Host Type	Parasite Name	Parasite Type	Region	Personality-Parasitism Correlation	Infection Metric	Study Type
23	*Turdus rubrocanus*	Aves	Plasmodium	Endoparasite	Asia	Boldness/Activity: no significant correlation	Infection rate	Correlational
24	*Luscinia megarhynchos*	Aves	Eimeria/Plasmodium	Endoparasite	Europe	Boldness/exploration: negative	Infection intensity	Correlational
25	*Ficedula albicollis*	Aves	Eimeria	Endoparasite	Europe	Boldness: no significant correlation	Infection rate	Correlational
26	*Parus major*	Aves	Plasmodium	Endoparasite	Europe	Boldness (♀: positive; ♂: no significant correlation)	Infection status	Correlational
27	*Parus major*	Aves	Tick	Ectoparasite	Europe	exploration: positive	Parasite load	Correlational
28	*Lagopus lagopus scoticus*	Aves	Nematode	Endoparasite	Europe	Aggression: negative	Infection status	Correlational
29	*Xanthocnemis zealandica*	Insecta	Trematode	Endoparasite	Oceania	Boldness/Activity: no significant correlation	Parasite load	Correlational
30	*Pyrrhocoris apterus*	Insecta	Mite	Ectoparasite	Europe	Boldness/Activity (♀: positive)	Infection status	Correlational
31	*Odontotaenius disjunctus*	Insecta	Nematode	Endoparasite	N. America	Boldness (♀: positive; ♂: negative)	Parasite load	Correlational
32	*Pimephales promelas*	Pisces	Trematode	Endoparasite	N. America	Boldness: positive; Activity: negative	Parasite load	Correlational
33	*Phoxinus*	Pisces	Trematode	Endoparasite	Europe	Activity: positive; exploration: no	Infection	BACI experiment

Study ID	Host Name	Host Type	Parasite Name	Parasite Type	Region	Personality-Parasitism Correlation	Infection Metric	Study Type
						significant correlation	intensity	
34	*Phoxinus*	Pisces	Trematode	Endoparasite	Europe	Activity: negative	Parasite load	Correlational
35	*Salmo trutta*	Pisces	Pearl mussel larvae	Ectoparasite	Europe	Activity: positive	Infection status	Correlational
36	*Salmo trutta*	Pisces	Tapeworm	Endoparasite	Europe	Boldness: no significant correlation	Parasite load	Correlational
37	*Oncorhynchus mykiss*	Pisces	Trematode	Endoparasite	Europe	Boldness: negative	Parasite load	BACI experiment
38	*Oncorhynchus mykiss*	Pisces	Trematode	Endoparasite	Not specified	Boldness/Activity: no significant correlation	Infection rate	Correlational
39	*Lepomis gibbosus*	Pisces	Trematode/Tapeworm	Endoparasite	N. America	Boldness: positive; Activity: negative	Parasite load	BACI experiment
40	*Poecilia reticulata*	Pisces	Monogenean	Ectoparasite	N. America	Boldness (♀: negative)	Parasite load	Correlational
41	*Gasterosteus aculeatus*	Pisces	Tapeworm	Endoparasite	Europe	Boldness/Activity/sociality: positive	Parasite biomass	BACI experiment
42	*Gasterosteus aculeatus*	Pisces	Microsporidia	Endoparasite	Europe	Boldness: negative	Infection status	Correlational
43	*Gobiomorphus cotidianus*	Pisces	Trematode	Endoparasite	Oceania	Aggression: positive	Parasite load	Correlational
44	*Gobiomorphus*	Pisces	Trematode	Endoparasite	Oceania	Boldness/Activity/Exploration/Aggression: no	Parasite load	Correlational

Study ID	Host Name	Host Type	Parasite Name	Parasite Type	Region	Personality-Parasitism Correlation	Infection Metric	Study Type
	cotidianus					significant correlation		
45	*Perca fluviatilis*	Pisces	Trematode/Tapeworm	Endoparasite	Europe	Aggression: negative	Infection intensity	Correlational
46	*Pimephales promelas*	Pisces	Trematode	Endoparasite	N. America	Boldness: no significant correlation	Parasite load	Correlational
47	*Oncorhynchus mykiss*	Pisces	Trematode	Endoparasite	Europe	Aggression: positive	Infection status	Correlational
48	*Neogobius melanostomus*	Pisces	Trematode	Endoparasite	Europe	Boldness: no significant correlation	Infection intensity	Correlational
49	*Faxonius rusticus*	Crustacea	Trematode	Endoparasite	N. America	Boldness: positive; Exploration: negative	Parasite load	Correlational
50	*Orconectes virilis*	Crustacea	Trematode	Endoparasite	Europe	Boldness/Aggression:positive	Parasite load	Correlational
51	*Faxonius rusticus*	Crustacea	Trematode	Endoparasite	Oceania	Boldness:positive; Exploration: negative	Infection intensity	Correlational
52	*Littorina littorea*	Gastropoda	Trematode	Endoparasite	Europe	Boldness: negative	Infection status	Correlational
53	*Lithobates sylvaticus*	Amphibia	Trematode	Endoparasite	Not specified	Activity: negative	Infection intensity	Correlational
54	*Rhinella marina*	Amphibia	Lungworm	Endoparasite	Oceania	Boldness/Activity: negative	Infection status	Correlational
